# Vibrationally-resolved RIXS reveals OH-group formation in oxygen redox active Li-ion battery cathodes†

**DOI:** 10.1039/d4cp01766h

**Published:** 2024-07-02

**Authors:** Moritz Hirsbrunner, Anastasiia Mikheenkova, Pontus Törnblom, Robert A. House, Wenliang Zhang, Teguh C. Asmara, Yuan Wei, Thorsten Schmitt, Håkan Rensmo, Soham Mukherjee, Maria Hahlin, Laurent C. Duda

**Affiliations:** a Condensed Matter Physics of Energy Materials, Division of X-ray Photon Science, Department of Physics and Astronomy, Uppsala University Box 516 751 20 Uppsala Sweden laurent.duda@physics.uu.se; b Department of Chemistry – Ångström Laboratory, Uppsala University Box 523 751 20 Uppsala Sweden; c Department of Materials, University of Oxford Oxford OX1 3PH UK; d Swiss Light Source, Photon Science Division, Paul Scherrer Institut CH-5232 Villigen PSI Switzerland

## Abstract

Vibrationally-resolved resonant inelastic X-ray scattering (VR-RIXS) at the O K-edge is emerging as a powerful tool for identifying embedded molecules in lithium-ion battery cathodes. Here, we investigate two known oxygen redox-active cathode materials: the commercial Li_*x*_Ni_0.90_Co_0.05_Al_0.05_O_2_ (NCA) used in electric vehicles and the high-capacity cathode material Li_1.2_Ni_0.13_Co_0.13_Mn_0.54_O_2_ (LRNMC) for next-generation Li-ion batteries. We report the detection of a novel vibrational RIXS signature for Li-ion battery cathodes appearing in the O K pre-peak above 533 eV that we attribute to OH-groups. We discuss likely locations and pathways for OH-group formation and accumulation throughout the active cathode material. Initial-cycle behaviour for LRNMC shows that OH-signal strength correlates with the cathodes state of charge, though reversibility is incomplete. The OH-group RIXS signal strength in long-term cycled NCA is retained. Thus, VR-RIXS offers a path for gaining new insights to oxygen reactions in battery materials.

## Abstract 2

Vibrational RIXS signatures reveal the formation of OH-groups in Li-ion battery cathodes.

## Graphical abstract

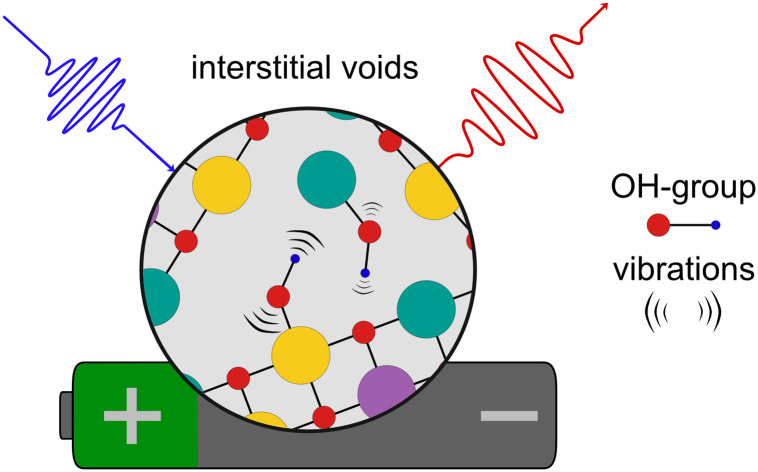

## 1 Introduction

Li-ion batteries have emerged as the leading choice for commercial energy storage across a broad spectrum of applications, spanning from portable electronics to electric vehicles and large-scale grid energy storage systems. Nonetheless, the capacity of the battery cathode remains a limiting factor in maximizing overall battery capacity. While reports of innovative Li-ion battery cathode materials with higher initial capacities are steadily emerging,^1^ most of these materials are plagued by aging effects that lead to a substantial loss of capacity over time, severely impacting their performance and service life.^2^ Understanding the mechanisms underlying battery aging in Li-ion batteries is imperative for enhancing their durability, reliability, and unleashing their full potential for clean energy storage. Layered transition metal oxides represent a promising class of materials for use as Li-ion battery cathodes. Notably, LiNi_*x*_Co_*y*_Mn_*z*_O_2_ is a crucial subcategory of materials employed in commercial Li-ion batteries, showcasing robust electrochemical properties and cycling stability that can be tailored through composition adjustments. Similarly, LiNi_*x*_Co_*y*_Al_*z*_O_2_ serves as a high-performance Li-ion battery cathode material that has been commercialized and is utilized in the Tesla Model 3 battery pack. Recent findings have demonstrated the substantial oxygen redox activity of this battery cathode material at both high potentials^3^ but also at surprisingly low potentials.^4^

Using vibrationally-resolved resonant inelastic X-ray scattering (VR-RIXS), House *et al.*^5^ concluded that oxygen redox cathodes effectively convert some of their lattice oxygen into molecular oxygen during the delithiation process. Interestingly, these oxygen molecules are not expelled from the crystal structure; instead, they become trapped within the crystals and appear to be reversibly integrated into the lattice upon cathode re-lithiation.^5,6^ The discovery is particularly intriguing as it strongly suggests that oxygen redox results in the generation of embedded species that are extrinsic to the oxide sublattice. This was not foreshadowed by Raman spectroscopy studies or photoelectron spectroscopy, instead, identification of these embedded oxygen molecules was enabled by the application of VR-RIXS,^5^ whereas X-ray absorption spectroscopy (XAS) alone is insufficient to unambiguously detect their presence due to the dominating signal of lattice oxygen. Remarkably, the signatures of these trapped oxygen molecules resemble gas-phase oxygen K-RIXS spectra,^5^ raising questions about their location, interactions with lattice atoms, and their stability over time.^6^ Although we will not go into further detail regarding this topic, we note that it has been shown that similar RIXS signatures may arise in certain non-Li-excess cathodes even in the absence of other evidence for oxygen redox.^7^ However, this observation only adds to the urgency for developing a picture of embedded molecules in Li ion cathodes, in general.

Another direction of inquiry concerns the formation of other oxygen species during synthesis or cycling and their identification using VR-RIXS. For instance, carbonates and intercalated water may typically be formed in battery cathodes, with identifiable signatures at energies above the X-ray absorption pre-peak region.^8^ Debates also persist about the formation and the impact of hydroxides on battery operation.^9^ These compounds are generated and evolve during battery cycles, especially in Ni-rich materials exposed to air or moisture. The mechanisms behind long-term cycling degradation and a comprehensive understanding of chemistry within Li-ion battery cathodes remain incomplete but are essential for addressing efficiency bottlenecks.

When several oxygen species are present in a material, disentangling their main band RIXS spectra can become very difficult. On the other hand, their vibrational signatures are often distinguishable and could therefore bring more clarity. High-resolution RIXS stands out as an exceptional tool for gaining detailed insights into the electronic structure changes within materials. Importantly, unlike in other core level spectroscopy techniques, RIXS spectra are not restricted by the core hole's lifetime. As such, it has been successfully employed to discern vibrational contributions in both molecules and condensed materials, which are typically investigated using Raman or IR-spectroscopy.^10–13^ Thus, when coupled with high-resolution synchrotron radiation, RIXS allows for the resolution of transitions originating from individual molecular vibrations. Though this capability is akin to optical Raman spectroscopy, VR-RIXS has the added advantage of elemental specificity, particularly for distinguishing contributions from oxygen and transition metal sites. Therefore, VR-RIXS is a powerful tool for advancing the understanding of oxygen redox processes in Li-ion battery cathodes.

In this study, we present a novel observation in O K-RIXS from hydroxide compounds forming in the oxygen redox active materials Li_1.2_Ni_0.13_Co_0.13_Mn_0.54_O_2_, a Li-rich, Mn-based NMC compound, and Li_*x*_Ni_0.90_Co_0.05_Al_0.05_O_2_, a Ni-rich NCA active material from commercial electrodes. LRNMC has a high initial capacity but suffers from capacity loss due to structural reformation after the initial cycle while NCA is structurally more stable making it already commercially viable. Considering their differences in composition and performance, interestingly, both display O_2_- and OH-formation.

Employing VR-RIXS, we unveil, for the first time, the vibrational progressions of these hydroxide groups in battery cathode materials. Our investigation reveals the occurrence of various vibrational modes of OH-groups, whose intensities are sensitive to the type of material and the state of charge. We discuss whether the observed OH-vibrations originates from compounds in the cathode electrolyte interphase (CEI) or from the bulk of the cathode particles. Suggestions on the formation pathways of the OH-groups are also presented. Furthermore, we track the evolution of these OH-groups during the initial cycle and observe changes in OH concentration during the aging process. In a broader context, this study underscores the chemical specificity of RIXS, which is achieved through energy-selective excitation within the O K-edge, resulting in distinct VR-RIXS signatures from various oxygen sites within the examined material.

## 2 Experimental

### 2.1 Sample preparation

The present study investigates Li_1.2_Ni_0.13_Co_0.13_Mn_0.54_O_2_ (LRNMC) which were prepared through sol–gel synthesis according to the procedure stated by House *et al.* (2020).^5^ The LRNMC samples were exclusively sourced from the initial charge/discharge cycle. The LRNMC sample series is based upon extracting electrochemically cycled cathodes of different lithium ion concentration (*i.e.* degree of lithiation) from batteries. It is composed of pristine, beginning of plateau (BoP), end of plateau (EoP), delithiated and re-lithiated samples. The plateau in BoP and EoP refers to the voltage plateau in the charge curve for LRNMC. The initial charge/discharge curve for LRNMC can be seen in Fig. S5 (ESI†), with demarcations of the corresponding voltage intervals from which the LRNMC samples were obtained. The LRNMC samples were sealed in pouches under Ar-atmosphere and rested for four years before the measurements.

The present study also investigates Li_*x*_Ni_0.90_Co_0.05_Al_0.05_O_2_ (NCA) in addition to the LRNMC. Note that the preparation of the NCA samples of the present study has already been described in a recent publication.^4^ For convenience, we also present relevant details in the following. The sample preparation of the NCA electrodes for the current study consisted of: (i) opening the full cylindrical cell and extracting the electrodes; (ii) reassembling the extracted electrodes into half-cells; (iii) charging the half-cells until reaching the desired state of charge (SOC) for further RIXS investigation. The full cells were extracted from commercial 21 700 cylindrical cells originating from a Tesla Model 3 2018 battery pack. Cells extracted from the pack were considered beginning of life. However, such cells have been pre-cycled at the factory. Two additional cells were aged at 45 °C by cycling between 0–50% SOC (resulting samples are called ‘aged 0–50’) and 0–100% SOC (named ‘aged 0–100’). Here, 0% SOC constitutes a voltage of 2.55 V, 50% SOC corresponds to 3.6 V and 100% SOC corresponds to 4.2 V. The charge and discharge curves for each sample are presented in Fig. S4 (ESI†). The cells achieved 950 (aged 0–50) and 1050 (aged 0–100) full equivalent cycles until reaching ∼20% capacity loss, which was considered the end of life for the aged cells. After, the cells were disconnected and discharged for further disassembly in the Ar-filled glove box. The central rectangular region of the cathode material was selected as the region of interest for the electrode extraction. After taking the central regions from all cells, the electrodes were mechanically cleaned from one side of the active material and punched with a diameter of 8 mm. The resulting electrodes were reassembled in pouch cells using pouch material (Skilstuna Flexible, pre-dried at 60 °C in an oven) with Li foil (thickness 450 μm, used as received) as a counter electrode, Celgard 2325 (Celgard, cleaned with ethanol, deionized water and dried in Buchi oven at 60 °C for 12 hours) as a separator, and LP40 (1 mol L^−1^ lithium hexafluorophosphate) dissolved in 1 : 1 v/v Ethylene carbonate: Diethyl carbonate (LP40, 1 M LiPF_6_ in 1 : 1 EC:DEC, Solvionic, used as received) was used as electrolyte. Pouch cells were cycled until the required potential using Arbin and Biologic cycling equipment. The assembled half-cells were first put at rest for 6 h, then discharged to 2.55 V and then cycled with a constant current of 50 μA until reaching the required SOC. After that, a constant voltage was applied until the current was lower than C/50. After, the cells were disassembled in an Ar-filled glovebox. A 2 mm strip was cut from every electrode and was placed on a sample holder for further RIXS studies.

### 2.2 X-ray spectroscopy

High resolution RIXS spectra of the oxygen K-edge were obtained at the soft X-ray beamline ADRESS at the Swiss Light Source, Villigen-PSI, Switzerland.^14^ RIXS measurements were conducted using the variable line density spherical grating (VLS) based Super Advanced X-ray Emission Spectrometer (SAXES) allowing for an energy range of 0.3 keV–1.6 keV and a resolution of 10 000, *i.e.* 50 meV at 500 eV. A scattering angle of 135° was used. To prevent exposure to air, samples were transported to the beamline in air-tight, evacuated aluminum pouches. The samples were transferred into the experiment chamber by attaching a glove bag to the load lock system, ensuring a safe atmosphere by purging the glove bag multiple times with argon. The glove bag was then kept at an overpressure under constant argon flow in order to prevent air from leaking into the system while the pouches were opened and the sample holders were transferred to the load lock. The O K-edge RIXS maps are made up of 36 RIXS spectra, each with an acquisition time of three minutes (two minutes of irradiation, one minute of dark time read-out). Although no beam damage was detected during prolonged irradiation, any possible damage to the battery samples was mitigated by sample translation to a fresh spot after each RIXS spectrum.

## 3 Results and discussion

### OH-group signatures in RIXS

**Fig. 1:**
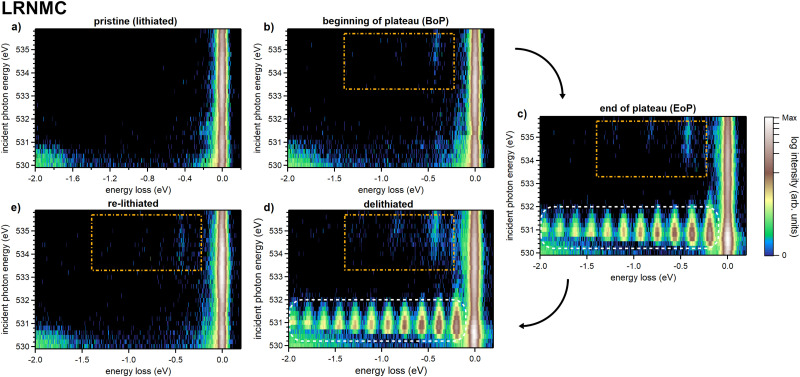
O K-edge RIXS maps of the initial cycle LRNMC samples in (a) pristine (lithiated), (b) beginning of plateau (BoP), (c) end of plateau (EoP), (d) delithiated and (e) re-lithiated. The orange dashed rectangle highlights the area in which vibrational progressions of an OH-group can be observed. At lower excitation energies, the white dashed box signifies the area where O_2_ signatures can be observed.

We have obtained high-resolution O K-edge RIXS maps of initial cycle LRNMC battery cathodes at various points along the charge/discharge curve. Here, we analyze the vibrational signatures observed for incident energies on the high energy side of the O K pre-peak, *i.e.* above ∼533 eV, a region that has not been focused on with VR-RIXS before. Fig. 1 shows O K-edge RIXS maps of the low energy loss region (−2 eV to 0.2 eV) of the five LRNMC samples. The elastic peak, which does not experience any energy loss, forms a continuous high-intensity band around 0 eV energy loss. Further, we point out the long progression of highly intense peaks belonging to O_2_-molecules in the delithiated cathodes (white dashed boxes), which have been discussed in detail by House *et al.*^5^

Now, we turn to the features highlighted by the orange dashed boxes at incident photon energies above ∼533 eV. Two peaks in this region can easily be identified by visual inspection of the RIXS maps. The lowest energy loss peak appears at an energy loss of ∼0.4 eV and a second, weaker peak at ∼0.8 eV. These features are representative of a vibrational progression which can be assigned to vibrational modes of an OH-group (described in detail further below). Note that this OH-signal is absent in the pristine LRNMC, indicating that the OH is formed during charge.

**Fig. 2:**
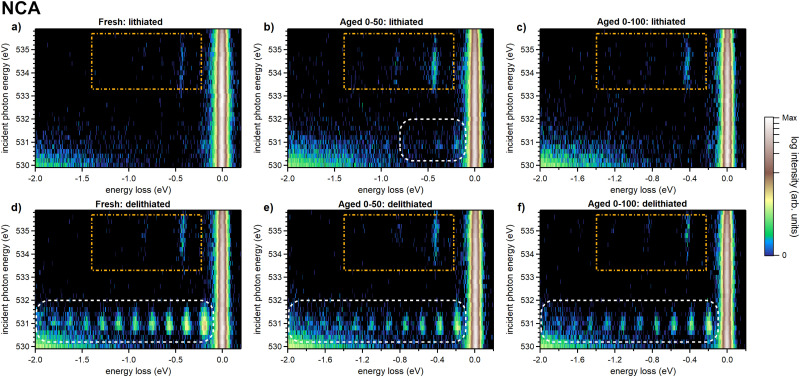
O K-edge RIXS maps of the lithiated (a)–(c) and delithiated (d)–(f) NCA samples in fresh, aged 0–50 and aged 0–100 conditions. The orange dashed rectangle highlights the area in which vibrational progressions of an OH-group can be observed. At lower excitation energies, the white dashed box signifies the area where O_2_ signatures can be observed.

High-resolution O K-edge RIXS maps of commercial automotive grade NCA battery cathodes have also been obtained and can be seen in Fig. 2. This sample series includes samples in both lithiated (discharged) and delithiated (charged) states from fresh, aged 0–50, and aged 0–100 NCA. Similarly to the LRNMC, the RIXS maps of the delithiated NCA in Fig. 2 display the O_2_-signal (white dashed boxes) which has been discussed by Mikheenkova *et al*.^4^

In the delithiated LRNMC and NCA samples, the intensity is an order of magnitude weaker than the corresponding O_2_-signal. We find that both the O_2_ and the OH-group signal are more intense (when at their strongest) in LRNMC compared to NCA when normalizing to the main band features at the respective excitation energies (Fig. S1, ESI†). Interestingly, the RIXS maps of NCA show a consistent absorption energy shift of the OH signal depending on the state of charge and we can infer the same tendency from the LRNMC RIXS maps, see Fig. S3 (ESI†). Both display a shift towards (higher) lower energies when (de)lithiated, indicating that the chemical environment of the OH-group changes with the state of charge.

**Fig. 3:**
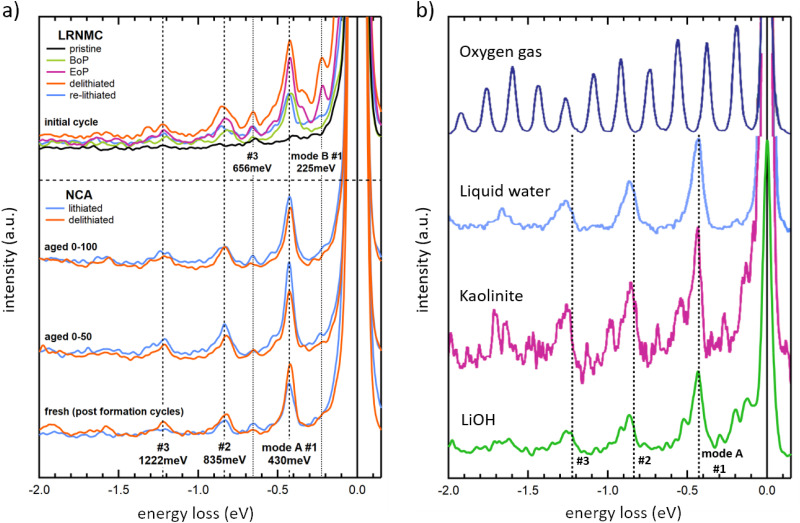
O K-edge RIXS spectra in the region of OH vibrations are shown. (a) Initial cycle LRNMC samples: pristine (lithiated), delithiated, beginning of plateau (BoP), end of plateau (EoP) and (re-)lithiated. On the bottom, fresh, aged 0–50 and aged 0–100 NCA samples in both delithiated and lithiated states are shown. The displayed data is derived by integrating over the RIXS spectra from 533.4 eV to 535.6 eV incident photon energy. A binomial smoothing over 14 channels (63 meV) was applied to all spectra. (b) Reference O K-edge RIXS vibrational signals are shown for oxygen gas, liquid water and Kaolinite (Al_2_Si_2_O_5_(OH)_4_), adapted from Schreck *et al.*, 2016,^15^ as well as LiOH (powder sample, this work).

We analyze the OH-signal from the RIXS maps further by creating 1D energy loss spectra, shown in Fig. 3a. The shown integrated spectra help clarify the observed peak structure whose energy positions can be more accurately determined. The displayed data is derived from the RIXS maps in Fig. 1 and 2 by integrating RIXS spectra along the incident photon energy axis in the range between 533.4 eV and 535.6 eV, combining them into a single spectrum. From this, we achieve a clear picture of the vibrational features for each sample. To enhance clarity, a binomial smoothing of 14 channels (63 meV) was applied to the spectra, which does not significantly reduce the experimentally attained resolution. The corresponding raw data can be found in the ESI† in Fig. S2.

For the LRNMC samples, a significantly smaller (larger) peak intensity of lithiated (delithiated) material is evident from the data presented in Fig. 3a, suggesting partially reversible behaviour. To a lesser degree this is also the case for the cycle of “fresh” NCA cathodes. We recall that “fresh” does not correspond to the very first cycle, so that the presence of a signal in lithiated NCA is consistent with the incomplete reversibility observed for cycled LRNMC. For the aged NCA samples, the OH-peak intensity stays at a high level regardless of lithiation, suggesting some degree of accumulation of OH during aging.

In the integrated spectra, a third peak is evident as well as an intricate substructure. For instance, close inspection of the peak at ∼430 meV reveals that the position noticeably shifts towards a lower energy loss position from the lithiated to the delithiated samples in both LRNMC and NCA. Additionally, for delithiated LRNMC, the 430 meV peak appears to be composed of multiple peaks. Providing an accurate identification of these features is outside of the scope of the present work and future improved resolution will certainly reveal more details. Here, we focus on the peaks belonging to two vibrational progressions. Firstly, the strongest progression is composed of a series of peaks at about 430 meV, 835 meV and 1222 meV (denoted as mode A #1, #2, #3 in the bottom half of Fig. 3a). We observe the Δ*E* between peaks decreasing for consecutive overtones from 430 meV to 405 meV and then to 387 meV. Furthermore, two peaks at 225 meV and 656 meV can be seen which belong to #1 and #3 of mode B, another vibrational progression which is most apparent in LRNMC. Peak #2 of this progression coincides with peak #1 of mode A, making it imperceptible. Below we discuss the origin of these progressions.

Vibrational excitations of stretching modes of OH groups have previously been observed between 3100 cm^−1^ (Δ*E* = 385 meV) and 3800 cm^−1^ (Δ*E* = 471 meV) using both experimental and theoretical techniques.^11,12,16–22^ The lower of these energy excitations have been related to more strongly-bound OH groups, while the largest Δ*E* can be assigned to free OH^−^ vibrations.^16,20,23^ Comparing this to the observed Δ*E* of mode A of 430 meV (3470 cm^−1^) suggests an assignment of these features to stretching mode vibrations of a bound OH-group. Strong vibrational progressions in O K-RIXS of liquid^15,19^ and gas phase water^24^ as well as from other compounds^15^ have been reported earlier. In Fig. 3b we show examples of compounds with similar signatures of previously reported O K-RIXS spectra from liquid water, kaolinite,^15^ and LiOH powder (this work). Additionally, the dashed lines indicate the energies taken from the vibrational progression mode A in Fig. 3a. We find that the observed main vibrational progressions of the references are similar with a somewhat larger spacing between the energy levels, as well as an intense low-energy structure that is markedly distinct from the OH-groups that we detect. The slight differences of the observed OH-signal in the cathode materials with the above reference compounds could be the result of a mixture of such OH-compounds, or it could mean that the vibrational frequency of the present OH-compound is altered because of its chemical environment. Note that, for free water molecules, both symmetric and asymmetric stretching vibrations would lie above 446 meV (3600 cm^−1^), which would suggest that the OH-groups are either bound to the structure directly in the form of a TM-OH, or there are water molecules which are restricted inside of the material structure. For reference, water can exhibit lower energy stretching vibrations in the range of 430 meV when hydrogen-bonded in complexes of four hydrogen molecules or more.^25^

In LRNMC, additional peaks at 225 meV and 660 meV belonging to a separate vibrational progression are apparent, while the NCA cathodes show weak hints of peaks at these energy losses (mode B in Fig. 3a). Tentatively, this could be attributed to bending modes (see gas phase water^24^) or other stretching modes^26^ (see also LiOH in Fig. 3b). On the other hand, bending mode energies of OH groups bound to transition metals are on the order of ∼50–80 meV (∼400–650 cm^−1^).^22^ Thus, the observation of different OH-peak mode intensities in LRNMC and NCA suggests that the OH-group surroundings are at least distinct in the two compounds, if not signifying completely different chemistries, which is corroborated by the richer substructure around the 430 meV peak of LRNMC.

In the studied NCA samples, OH-groups were found in both fresh and aged materials in both the lithiated and the delithiated states (see Fig. 3a). While the presence of OH-vibrations in the fresh NCA samples could be interpreted as originating from OH-groups that are produced during the material synthesis, we explain in the following why we deem this to be unlikely. The presence of OH-groups in Ni-rich materials has been established in previous studies using other techniques.^27–34^ Hydroxide compounds can be found in transition metal oxide materials, such as NCA, at different stages of the materials production (the detailed description of the formed and consumed species is provided in the ESI†). However, as mentioned in the ESI,† there should, at most, only be trace amounts of OH-containing compounds concentrated at the surface of the NCA from the material production. Detection of these trace amounts with the used experimental parameters is unlikely. Therefore, we believe that the observed OH signal is related to OH-groups that formed by cycling, which is supported by the fact that an OH signal is absent in the pristine LRNMC sample. Thus, we conclude that the observed signal is not a result of remnant precursors inside of the samples.

### Possible formation pathways

In the following, we discuss whether the OH-containing compounds are located in the CEI or the bulk of the cathodes. RIXS, in the present energy range, has a typical mean information depth of 100 nm, which strongly favors signals from bulk layers or internal interfaces (if present) over that from the surface. The observed signal could stem from a concentrated stratification of OH-compounds as in CEI formation, or from a distribution of formed OH-compounds like TM-OH in the bulk of the active material. The former appears less likely due to the observed CEI thickness of only a few nanometers^35–37^ compared to the size of the primary particles (∼100 nm) and secondary particles (5–15 μm) in the case of NCA.^4^ In addition, internal interfaces are not enhanced by particle cracks as those are not prevalent during the initial cycles as observed by our previous study on NCA.^4^ However, the formed CEI could contribute significantly to the RIXS signal if the concentration of OH-groups is sufficiently high. All OH-formation pathways proposed here require H^+^-formation at the surface of the secondary particle which would suggest that the resulting OH-groups are formed and concentrated at the surface. Although commonly employed surface sensitive techniques like photoelectron spectroscopy rarely find traces of OH-groups forming in the CEI, the signal can be hard to isolate as it overlaps with carbonate contributions.^29,38^ Nevertheless, theory supports H^+^- and OH-formation on the surface of different cathode materials^39,40^ due to ethylene carbonate (EC) electrolyte decomposition, and it is suggested that high nickel content could promote this process.^39^ This is further discussed in ESI† Section S1. Residues from electrolyte after battery disassembly, which could also add to the observed OH-signal, are removed by carefully rinsing the samples with dimethyl carbonate (DMC) and drying them.

One possible OH-formation pathway could stem from the proton exchange mechanism which has been reported for Ni-rich materials.^31^ This effect can be observed both at the surface and in the bulk because protons can migrate into the active material structure,^41^ making the OH-groups formed through this mechanism the most relevant for RIXS measurements due to its bulk sensitivity. There are two ways of forming H^+^ in the cell. Both include degradation of the electrolyte at higher and lower potentials. First, the dehydrogenation of EC to VC (vinylene carbonate) can take place at lower potentials according to reaction (1).^42^ The reaction occurs at the surface of the cathode material. Since H^+^ has high mobility, there are significant chances of protonating the bulk of the active material.1
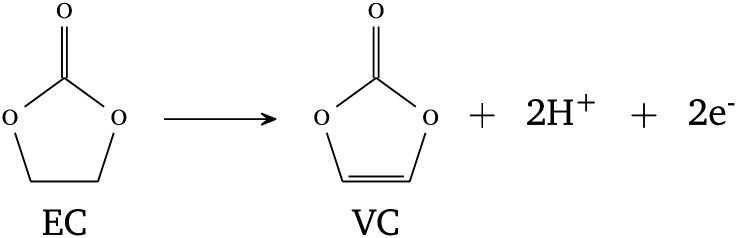


Another pathway of protonation includes degradation of the EC electrolyte as a result of oxidation at higher potentials.^33,43^ The mechanism is a two-step process. First, oxygen, previously shown to be formed within the LRNMC and NCA structure, can move to the interface where it oxidizes EC electrolyte according to reaction (2):^43^2
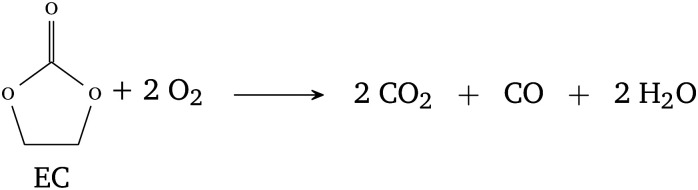


Support for this reaction is the detection of CO_2_ when cycling at higher potentials which was measured on the same NCA electrodes.^44^ The generated water further reacts with Li^+^, which is present on the surface of the secondary particles^29,31^ and the following reaction occurs:

3 Li^+^ + H_2_O → H^+^ + LiOH

Reaction (3) takes place on the surface of the active material. Subsequently, the formed protons can diffuse into the active material and once H^+^ is inside of the cathode structure, it is likely to form OH groups (4):

4 LiTMO_2_ + H^+^ → LiTMO-OH

**Fig. 4:**
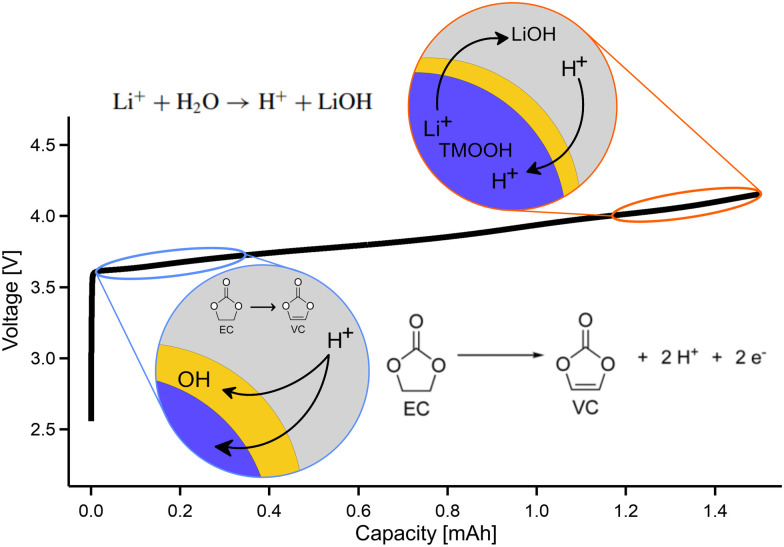
Schematic display of the chemical reactions responsible for H^+^ formation, occurring at low and high potentials, exemplified with the charging curve of NCA. The detailed reaction schemes can be found in the text.

These oxyhydroxides have been observed in Ni-rich NMC before^30^ and previous studies of oxyhydroxides have shown that they are stable up to 4.2 V *vs.* Li/Li^+^,^45^ which supports the hypothesis that the formation of OH-groups is partially irreversible in the working range of the material. However, note that, assigning the exact mechanism of OH formation inside the cathode is difficult and outside the scope of the current study. Fig. 4 shows a schematic depiction of the proton exchange mechanism in connection to the charging curve of NCA. Low potential electrolyte decomposition is shown in light blue while the high potential proton exchange mechanism is shown in orange. We point out that all the formation pathways mentioned above may be substantially assisted by internal lattice superstructures,^5^ nanoporosity^4^ or interstitial pores or voids from microstructural defects within the cathode particles.^46^

## 4 Conclusion

We present a novel type of O K-RIXS signature from the oxygen redox active compounds LRNMC and NCA that emerge during cycling alongside the previously reported trapped-O_2_ RIXS signals. We reveal for the first time that lithium-ion batteries exhibit vibrational progressions that can be attributed to stretching modes of OH-groups. The OH-group signal strength increases upon charging in the initial-cycle of LRNMC, which is consistent with indications from early-cycle NCA. However, in contrast to O_2_ formation, reversibility is incomplete in initial-cycle LRNMC and negligible for NCA, while the RIXS maps of both sample systems still show a consistent absorption energy shift of the OH signal depending on the state of charge. Our study shows that cathode materials are prone to develop OH-groups, which is an undesirable side reaction in contrast to O_2_ formation, which serves as a method for reversible energy storage. Aged NCA suggests that OH-group concentration stays fairly stable after a build-up during early cycles, and may even become more enhanced during lithiation. This observation points to the fact that the OH-formation plateaus and constitutes small amounts which should not strongly impede the cathode performance. While this is the case for the present cathodes, other battery chemistries might be affected more strongly and should be investigated. Besides the immediate observations for these cathode systems, this study highlights the power of vibrationally-resolved RIXS as a fingerprinting technique to identify characteristic embedded molecules and to study their surroundings. This is deemed especially important in understanding and troubleshooting the battery cathode chemistry. We believe this work can also stimulate the difficult and direly needed theoretical task of calculating energy surfaces and simulating RIXS spectra of embedded molecules. Our findings are thus instrumental in gaining a comprehensive understanding of the entire cathode system, one of the most topical questions in lithium-ion battery research.

## Author contributions

Moritz Hirsbrunner: formal analysis, investigation, project administration, validation, visualization, writing – original draft. Anastasiia Mikheenkova: formal analysis, resources (NCA), writing – original draft. Pontus Törnblom: formal analysis, investigation, validation, writing – original draft. Robert A. House: resources (LRNMC). Wenliang Zhang, Teguh C. Asmara, Yuan Wei: investigation. Thorsten Schmitt: investigation, instrumentation. Soham Mukherjee: investigation, supervision. Håkan Rensmo, Maria Hahlin: funding acquisition, supervision. Laurent Duda: conceptualization, funding acquisition, investigation, project administration, supervision, validation, writing – original draft. All authors participated in the writing and review of the manuscript.

## Data availability

All data related to this study is included in the figures contained in this article. Corresponding raw data is shown in the ESI.†

## Conflicts of interest

There are no conflicts to declare.

## Supplementary Material

CP-026-D4CP01766H-s001

CP-026-D4CP01766H-s002

CP-026-D4CP01766H-s003

CP-026-D4CP01766H-s004

CP-026-D4CP01766H-s005

CP-026-D4CP01766H-s006

CP-026-D4CP01766H-s007

## References

[cit1] Wang X., Ding Y. L., Deng Y. P., Chen Z. (2020). Adv. Energy Mater..

[cit2] Edge J. S., O'Kane S., Prosser R., Kirkaldy N. D., Patel A. N., Hales A., Ghosh A., Ai W., Chen J., Yang J., Li S., Pang M. C., Diaz L. B., Tomaszewska A., Marzook M. W., Radhakrishnan K. N., Wang H., Patel Y., Wu B., Offer G. J. (2021). Phys. Chem. Chem. Phys..

[cit3] Lebens-Higgins Z. W., Faenza N. V., Radin M. D., Liu H., Sallis S., Rana J., Vinckeviciute J., Reeves P. J., Zuba M. J., Badway F., Pereira N., Chapman K. W., Lee T. L., Wu T., Grey C. P., Melot B. C., Ven A. V. D., Amatucci G. G., Yang W., Piper L. F. (2019). Mater. Horiz..

[cit4] Mikheenkova A., Mukherjee S., Hirsbrunner M., Törnblom P., Tai C.-W., Segre C. U., Ding Y., Zhang W., Asmara T. C., Wei Y., Schmitt T., Rensmo H., Duda L., Hahlin M. (2024). J. Mater. Chem. A.

[cit5] House R. A., Rees G. J., Pérez-Osorio M. A., Marie J. J., Boivin E., Robertson A. W., Nag A., Garcia-Fernandez M., Zhou K. J., Bruce P. G. (2020). Nat. Energy.

[cit6] House R. A., Marie J. J., Pérez-Osorio M. A., Rees G. J., Boivin E., Bruce P. G. (2021). Nat. Energy.

[cit7] Menon A., Johnston B., Booth S., Zhang L., Kress K., Murdock B., Fajardo G. P., Anthonisamy N., Tapia-Ruiz N., Agrestini S., Garcia-Fernandez M., Zhou K., Thakur P., Lee T., Nedoma A., Cussen S., Piper L. (2023). PRX Energy.

[cit8] Genreith-Schriever A. R., Banerjee H., Menon A. S., Bassey E. N., Piper L. F., Grey C. P., Morris A. J. (2023). Joule.

[cit9] Li T., Yuan X. Z., Zhang L., Song D., Shi K., Bock C. (2020). Electrochem. Energy Rev..

[cit10] Yavaş H., Veenendaal M. V., Brink J. V. D., Ament L. J., Alatas A., Leu B. M., Apostu M. O., Wizent N., Behr G., Sturhahn W., Sinn H., Alp E. E. (2010). J. Phys.: Condens. Matter.

[cit11] Harada Y., Tokushima T., Horikawa Y., Takahashi O., Niwa H., Kobayashi M., Oshima M., Senba Y., Ohashi H., Wikfeldt K. T., Nilsson A., Pettersson L. G., Shin S. (2013). Phys. Rev. Lett..

[cit12] Harada Y., Miyawaki J., Niwa H., Yamazoe K., Pettersson L. G., Nilsson A. (2017). J. Phys. Chem. Lett..

[cit13] Rubensson J. E., Pietzsch A., Hennies F. (2012). J. Electron Spectrosc. Relat. Phenom..

[cit14] Strocov V. N., Schmitt T., Flechsig U., Schmidt T., Imhof A., Chen Q., Raabe J., Betemps R., Zimoch D., Krempasky J., Wang X., Grioni M., Piazzalunga A., Patthey L. (2010). J. Synchrotron Radiat..

[cit15] Schreck S., Pietzsch A., Kennedy B., Såthe C., Miedema P. S., Techert S., Strocov V. N., Schmitt T., Hennies F., Rubensson J. E., Föhlisch A. (2016). Sci. Rep..

[cit16] Lutz H. D., Eckers W., Haeuseler H. (1982). J. Mol. Struct..

[cit17] Savchenko V., Brumboiu I. E., Kimberg V., Odelius M., Krasnov P., Liu J. C., Rubensson J. E., Björneholm O., Såthe C., Gråsjö J., Dong M., Pietzsch A., Föhlisch A., Schmitt T., McNally D., Lu X., Polyutov S. P., Norman P., Iannuzzi M., Gel'mukhanov F., Ekholm V. (2021). Sci. Rep..

[cit18] Savchenko V., Ekholm V., Brumboiu I. E., Norman P., Pietzsch A., Föhlisch A., Rubensson J. E., Gråsjö J., Björneholm O., Såthe C., Dong M., Schmitt T., McNally D., Lu X., Krasnov P., Polyutov S. P., Gel'mukhanov F., Odelius M., Kimberg V. (2021). J. Chem. Phys..

[cit19] Pietzsch A., Hennies F., Miedema P. S., Kennedy B., Schlappa J., Schmitt T., Strocov V. N., Föhlisch A. (2015). Phys. Rev. Lett..

[cit20] Bernard M. C., Cortes R., Keddam M., Takenouti H., Bernard P., Senyarich S. (1996). J. Power Sources.

[cit21] Senthil Kumar N., Ganapathy M., Sharmila S., Shankar M., Vimalan M., Vetha Potheher I. (2017). J. Alloys Compd..

[cit22] Wang X., Andrews L. (2006). J. Phys. Chem. A.

[cit23] Okazaki S., Okada I. (1993). J. Chem. Phys..

[cit24] da Cruz V. V., Ertan E., Couto R. C., Eckert S., Fondell M., Dantz M., Kennedy B., Schmitt T., Pietzsch A., Guimarães F. F., Ågren H., Gel'mukhanov F., Odelius M., Föhlisch A., Kimberg V. (2017). Phys. Chem. Chem. Phys..

[cit25] Buck U., Huisken F. (2000). Chem. Rev..

[cit26] Higgins K. J., Freund S. M., Klemperer W., Apponi A. J., Ziurys L. M. (2004). J. Chem. Phys..

[cit27] You Y., Celio H., Li J., Dolocan A., Manthiram A. (2018). Angew. Chem..

[cit28] Liu H., Yang Y., Zhang J. (2006). J. Power Sources.

[cit29] Seong W. M., Kim Y., Manthiram A. (2020). Chem. Mater..

[cit30] Martinez A. C., Grugeon S., Cailleu D., Courty M., Tran-Van P., Delobel B., Laruelle S. (2020). J. Power Sources.

[cit31] Chen A., Wang K., Li J., Mao Q., Xiao Z., Zhu D., Wang G., Liao P., He J., You Y., Xia Y. (2020). Front. Energy Res..

[cit32] Cho D.-H., Jo C.-H., Cho W., Kim Y.-J., Yashiro H., Sun Y.-K., Myung S.-T. (2014). J. Electrochem. Soc..

[cit33] Renfrew S. E., McCloskey B. D. (2017). J. Am. Chem. Soc..

[cit34] Laurita A., Zhu L., Cabelguen P.-E., Auvergniot J., Guyomard D., Moreau P., Dupré N. (2023). Chem. Commun..

[cit35] Gao Z., Zhou K., Wu J., Kang F., Zhao C., Peng L., Li B. (2022). J. Phys. Chem. C.

[cit36] Lee S., Li W., Dolocan A., Celio H., Park H., Warner J. H., Manthiram A. (2021). Adv. Energy Mater..

[cit37] Yi M., Li W., Manthiram A. (2022). Chem. Mater..

[cit38] Chen H., Ericson T., Temperton R. H., Källquist I., Liu H., Eads C. N., Mikheenkova A., Andersson M., Kokkonen E., Brant W. R., Hahlin M. (2023). ACS Appl. Energy Mater..

[cit39] Østergaard T. M., Giordano L., Castelli I. E., Maglia F., Antonopoulos B. K., Shao-Horn Y., Rossmeisl J. (2018). J. Phys. Chem. C.

[cit40] Okuno Y., Ushirogata K., Sodeyama K., Shukri G., Tateyama Y. (2019). J. Phys. Chem. C.

[cit41] Shkrob I. A., Gilbert J. A., Phillips P. J., Klie R., Haasch R. T., Bareño J., Abraham D. P. (2017). J. Electrochem. Soc..

[cit42] Rinkel B. L., Vivek J. P., Garcia-Araez N., Grey C. P. (2022). Energy Environ. Sci..

[cit43] Jung R., Metzger M., Maglia F., Stinner C., Gasteiger H. A. (2017). J. Electrochem. Soc..

[cit44] Mikheenkova A., Gustafsson O., Misiewicz C., Brant W. R., Hahlin M., Lacey M. J. (2023). J. Energy Storage.

[cit45] Cheng L., Li H. Q., Xia Y. Y. (2006). J. Solid State Electrochem..

[cit46] Hu E., Yu X., Lin R., Bi X., Lu J., Bak S., Nam K. W., Xin H. L., Jaye C., Fischer D. A., Amine K., Yang X. Q. (2018). Nat. Energy.

