# Supplementary material for: Vibrationally-resolved RIXS reveals OH-group formation in oxygen redox active Li-ion battery cathodes
Source: Phys Chem Chem Phys. 2024 Jul 2;26(28):19460–8. doi: 10.1039/d4cp01766h (PMC11253246; doi:10.1039/d4cp01766h)
Supplement: CP-026-D4CP01766H-s002 [file CP-026-D4CP01766H-s002.pdf]

Electronic Supplementary Material (ESI) for Physical Chemistry Chemical Physics.
This journal is © the Owner Societies 2024

Supplemental information - Vibrationally-resolved RIXS reveals OH-group formation in oxygen redox active Li-ion battery cathodes

Moritz Hirsbrunner[a], Anastasiia Mikheenkova[b], Pontus Törnblom[a], Robert A. House[c], Wenliang Zhang[d], Teguh C. Asmara[d], Yuan Wei[d], Thorsten Schmitt[d], Håkan Rensmo[a], Soham Mukherjee[a], Maria Hahlin[a,b], and Laurent C. Duda[a]

[a]Condensed Matter Physics of Energy Materials, Division of X-ray Photon Science, Department of Physics and Astronomy, Uppsala University, Box 516, 751 20 Uppsala, Sweden
[b]Department of Chemistry - Ångström Laboratory, Uppsala University, Box 523, 751 20 Uppsala, Sweden
[c]Department of Materials, University of Oxford, Oxford OX1 3PH, United Kingdom
[d]Swiss Light Source, Photon Science Division, Paul Scherrer Institut, CH-5232 Villigen PSI, Switzerland

1 Surface OH-group Formation of Ni-rich Cathodes

First, LiOH or $Al(OH)_3$ are introduced as precursors. However, after high temperature treatment, which is applied as one of the last steps in synthesis, such compounds are usually left as oxides (Li_2O, for example)[1]. Therefore, OH is not expected to be detected in the material after the initial production. After the synthesis stage, the material typically comes into contact with the ambient atmosphere, which contains water and carbon dioxide. Contact with these particular compounds is found to be detrimental for the Ni-rich materials, as residual oxide impurities from the material surface can react with water and CO_2[2]. As a result of the reaction, LiOH is formed on the cathode material surface together with Li_2CO_3[3–6]. It is expected that any LiOH formed at ambient conditions is likely to be consumed with CO_2 resulting in Li_2CO_3[1,7], albeit that some studies show that the material can still contain LiOH[7]. Before the cell is released on the market, formation cycles are performed (several charge/discharge cycles at a slower rate). It is reported that during the formation cycles these residual lithium hydroxide and carbonate compounds are consumed. Some studies show that the compounds formed upon material synthesis and storage are one of the main sources of outgassing during the first cycle of the battery indicating that they are consumed during that cycle[8,9]. Additionally, the formation of OH-groups bonded to the transition metal of the active material upon low-potential electrolyte decomposition is a possible formation pathway for OH-groups. It results in the formation of oxyhydroxide at the surface of the material[10]. This means that such compounds will be formed at the places where the active material has contact with the electrolyte. However, RIXS is a bulk sensitive technique making it unlikely to detect such small amounts from a process that is connected to the surface.

2 RIXS Data

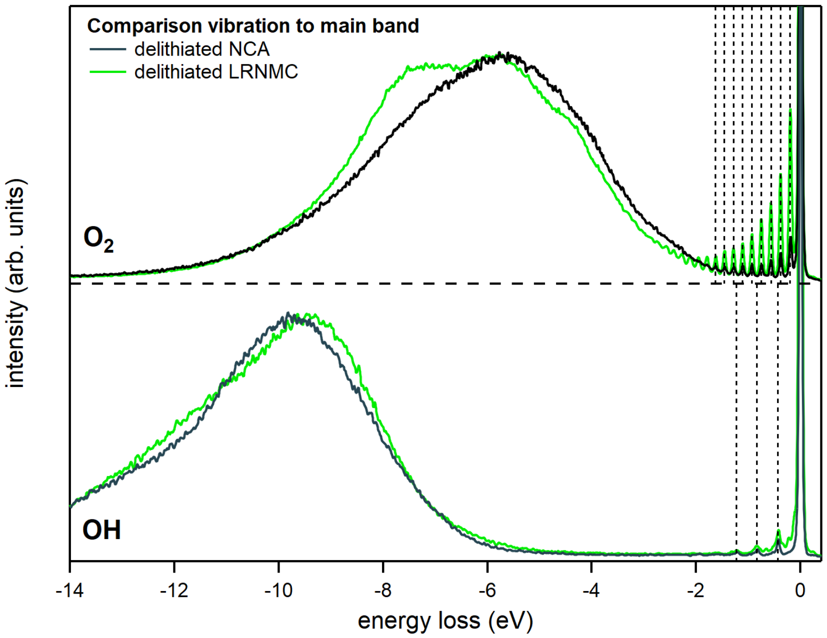

Fig. ESI 1. O *K*-edge RIXS spectra of delithiated LRNMC and NCA (fresh) are shown to compare the intensity of the vibrational feature of OH at low energy loss with the main band feature at roughly 10 eV energy loss. The spectra have been scaled to the same peak intensity of the main band feature. The displayed data is derived by integrating over the RIXS spectra from 533.4 eV to 535.6 eV excitation energy.

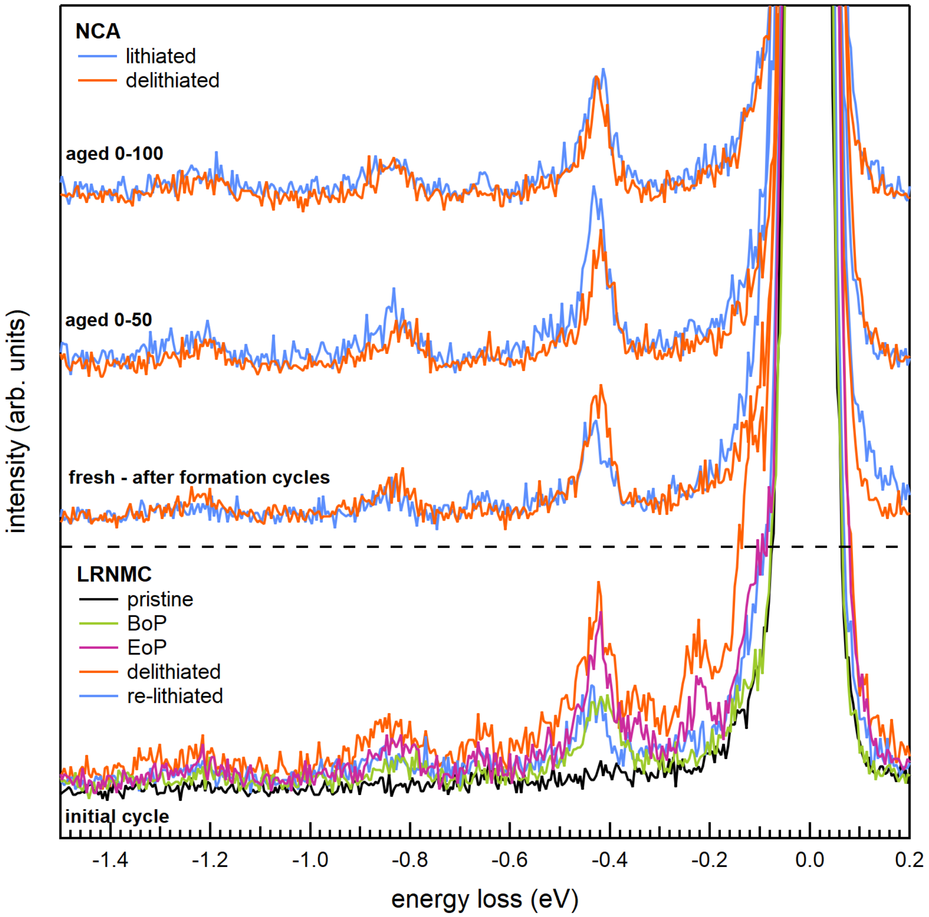

Fig. ESI 2. Unsmoothed O *K*-edge RIXS spectra in the region of OH vibrations are shown for NCA and LRNMC samples. The displayed data is derived by integrating over the RIXS spectra from 533.4 eV to 535.6 eV excitation energy.

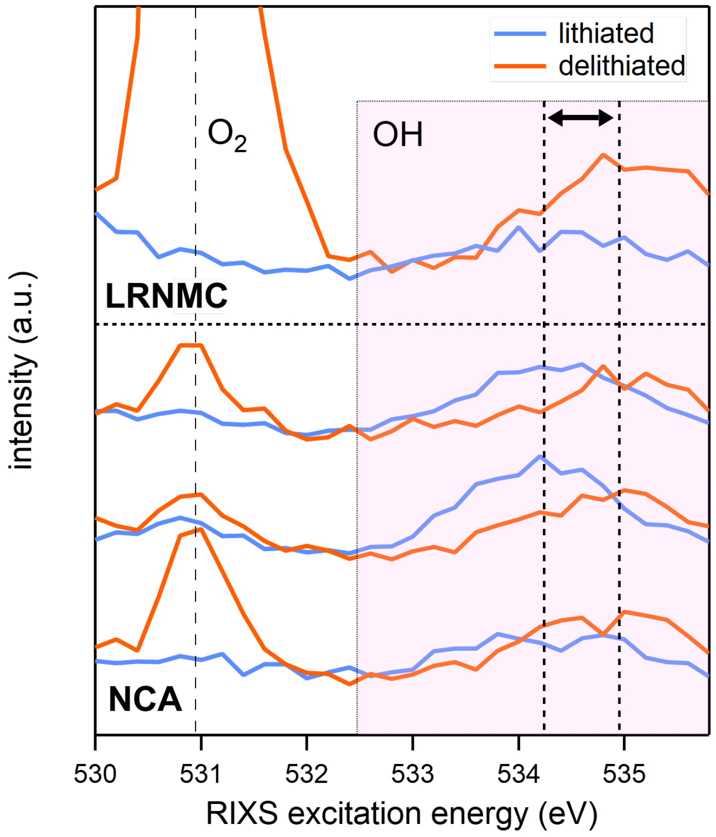

Fig. ESI 3. Partial RIXS yield (PRIXSY) of LRNMC and NCA samples. A shift in energy position of the OH peak can be observed between lithiated and delithiated samples. The spectra are displayed along the excitation energy axis and are made by integrating over the first OH-stretching mode peak from 0.387 eV to 0.478 eV RIXS energy loss (21 channels - 91meV). The vertical dashed lines are guides to the eye for the center of gravity of the O_2 and OH peaks. The double arrow indicates the shift of the OH peak during cycling.

3 Electrochemical Measurements

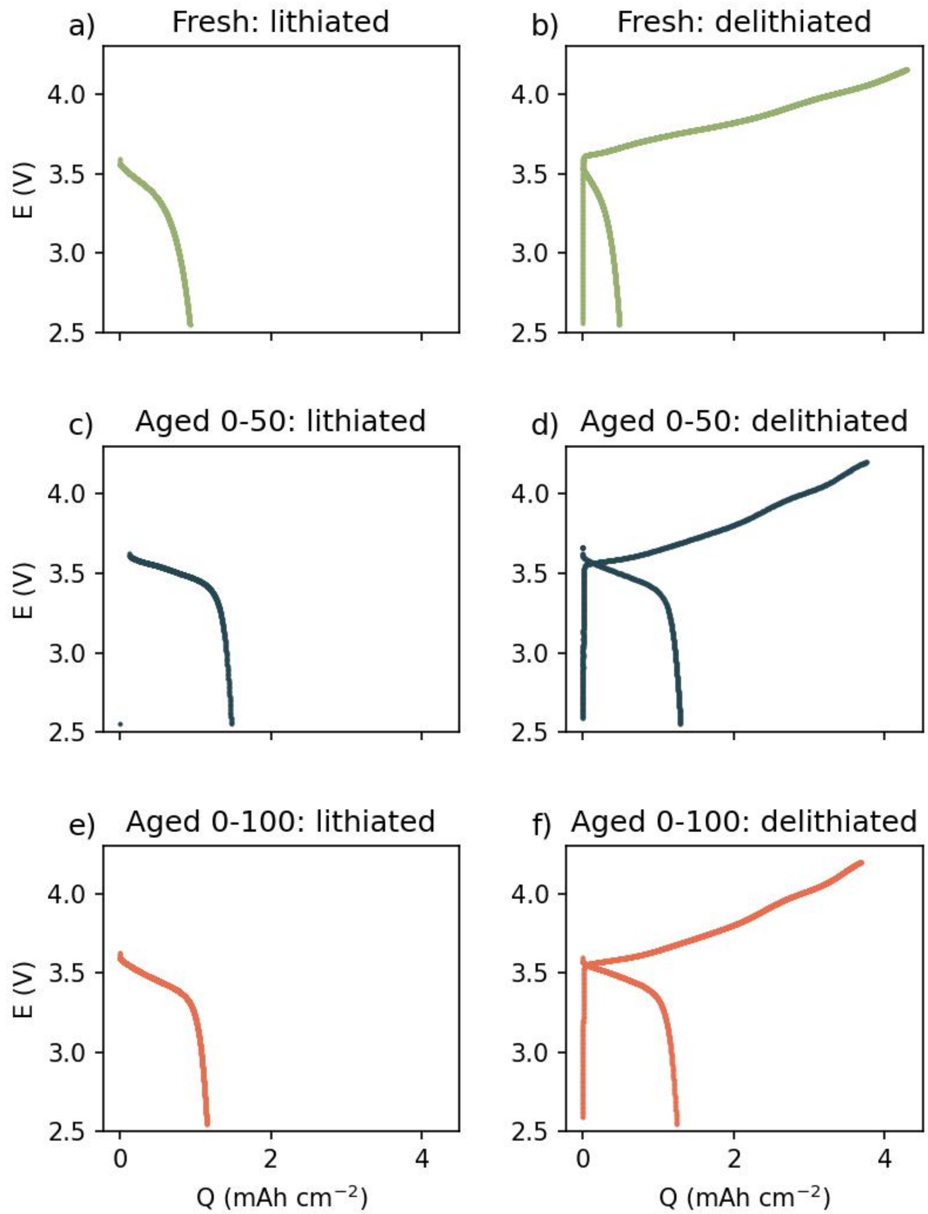

Fig. ESI 4. Initial charge/discharge curves for the NCA samples studied in the current work: (a) "Fresh: lithiated"; (b) "Fresh: delithiated"; (c) "Aged 0-50: lithiated"; (d) "Aged 0-50: delithiated"; (e) "Aged 0-100: lithiated"; (f) "Aged 0-100: delithiated".

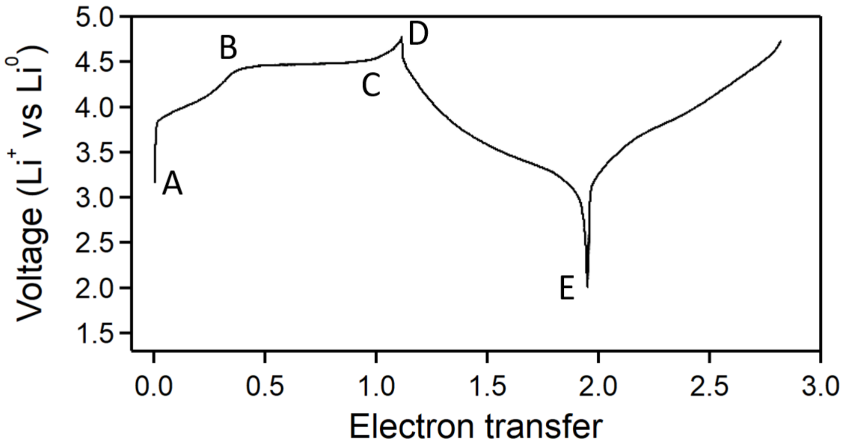

Fig. ESI 5. Charge/discharge curve for the LRNMC samples studied in the current work: (A) pristine; (B) beginning of plateau (BoP); (C) end of plateau (EoP); (D) delithiated; (E) re-lithiated.

References

[1] A. Chen, K. Wang, J. Li, Q. Mao, Z. Xiao, D. Zhu, G. Wang, P. Liao, J. He, Y. You and Y. Xia, *Frontiers in Energy Research*, 2020, **8**, 593009.

[2] C. Busà, M. Belekoukia and M. J. Loveridge, *Electrochimica Acta*, 2021, **366**, 137358.

[3] Y. You, H. Celio, J. Li, A. Dolocan and A. Manthiram, *Angewandte Chemie*, 2018, **130**, 6590–6595.

[4] H. Liu, Y. Yang and J. Zhang, *Journal of Power Sources*, 2006, **162**, 644–650.

[5] W. M. Seong, Y. Kim and A. Manthiram, *Chemistry of Materials*, 2020, **32**, 9479–9489.

[6] A. C. Martinez, S. Grugeon, D. Cailleu, M. Courty, P. Tran-Van, B. Delobel and S. Laruelle, *Journal of Power Sources*, 2020, **468**, 228204.

[7] D.-H. Cho, C.-H. Jo, W. Cho, Y.-J. Kim, H. Yashiro, Y.-K. Sun and S.-T. Myung, *Journal of The Electrochemical Society*, 2014, **161**, A920–A926.

[8] S. E. Renfrew and B. D. McCloskey, *Journal of the American Chemical Society*, 2017, **139**, 17853–17860.

[9] A. Laurita, L. Zhu, P.-E. Cabelguen, J. Auvergniot, D. Guyomard, P. Moreau and N. Dupré, *Chemical Communications*, 2023, **59**, 4951–4953.

[10] B. L. Rinkel, J. P. Vivek, N. Garcia-Araez and C. P. Grey, *Energy and Environmental Science*, 2022, **15**, 3416–3438.
