# Supplementary material for: Vibrationally-resolved RIXS reveals OH-group formation in oxygen redox active Li-ion battery cathodes
Source: Phys Chem Chem Phys. 2024 Jul 2;26(28):19460–8. doi: 10.1039/d4cp01766h (PMC11253246; doi:10.1039/d4cp01766h)

Electronic Supplementary Material (ESI) for Physical Chemistry Chemical Physics.
This journal is © the Owner Societies 2024

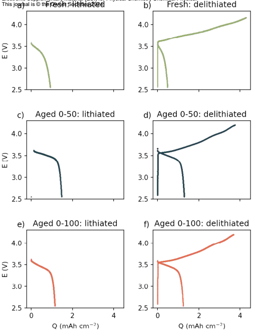

Supplement: CP-026-D4CP01766H-s003 [file CP-026-D4CP01766H-s003.pdf]
