# Supplementary figures and images for: Vibrationally-resolved RIXS reveals OH-group formation in oxygen redox active Li-ion battery cathodes
Source: Phys Chem Chem Phys. 2024 Jul 2;26(28):19460–8. doi: 10.1039/d4cp01766h (PMC11253246; doi:10.1039/d4cp01766h)

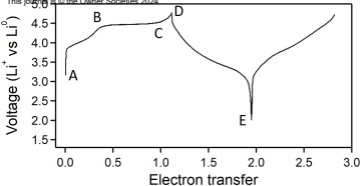

Supplement: CP-026-D4CP01766H-s004 [file CP-026-D4CP01766H-s004.pdf]

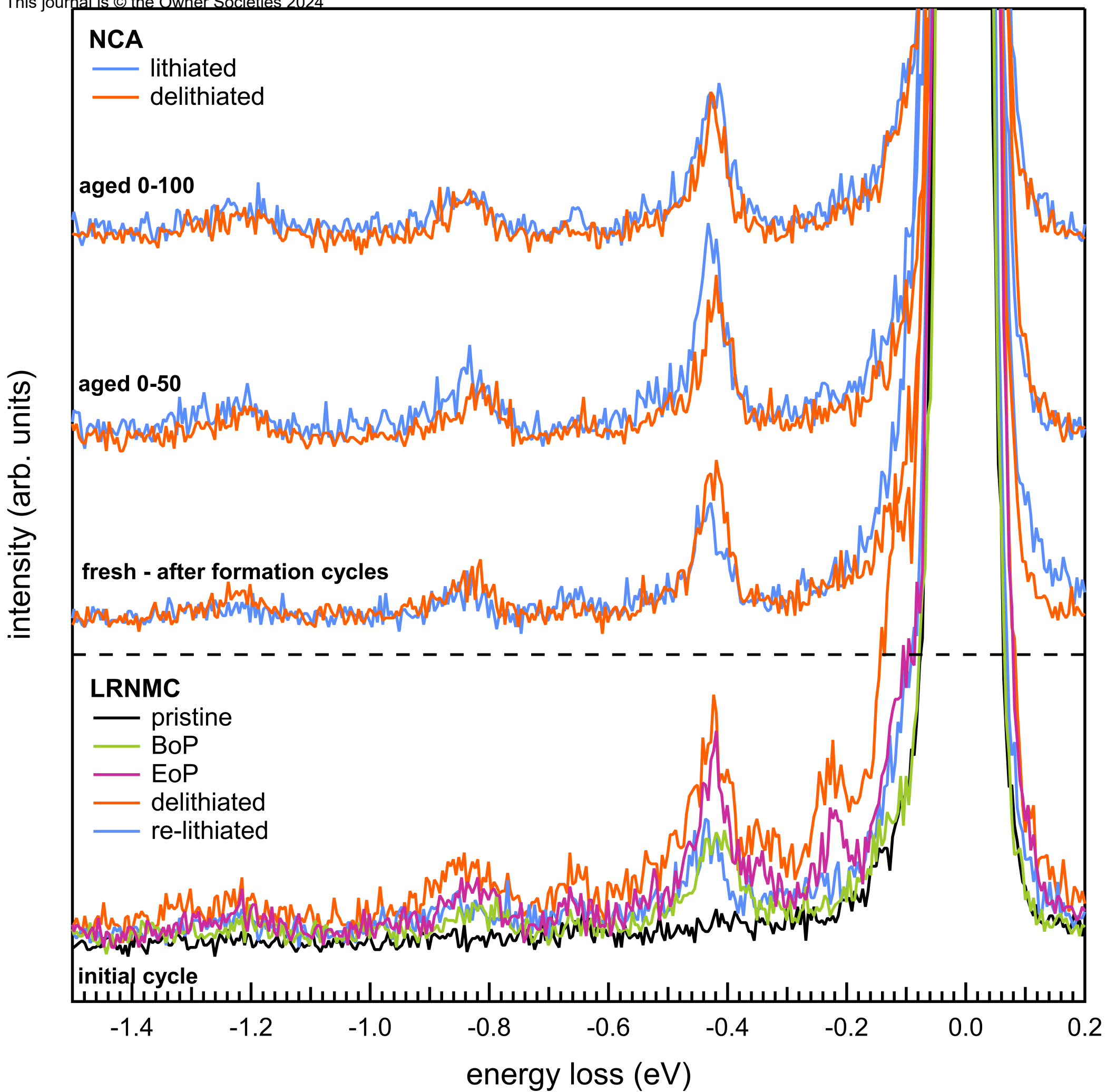

Supplement: CP-026-D4CP01766H-s005 [file CP-026-D4CP01766H-s005.pdf]

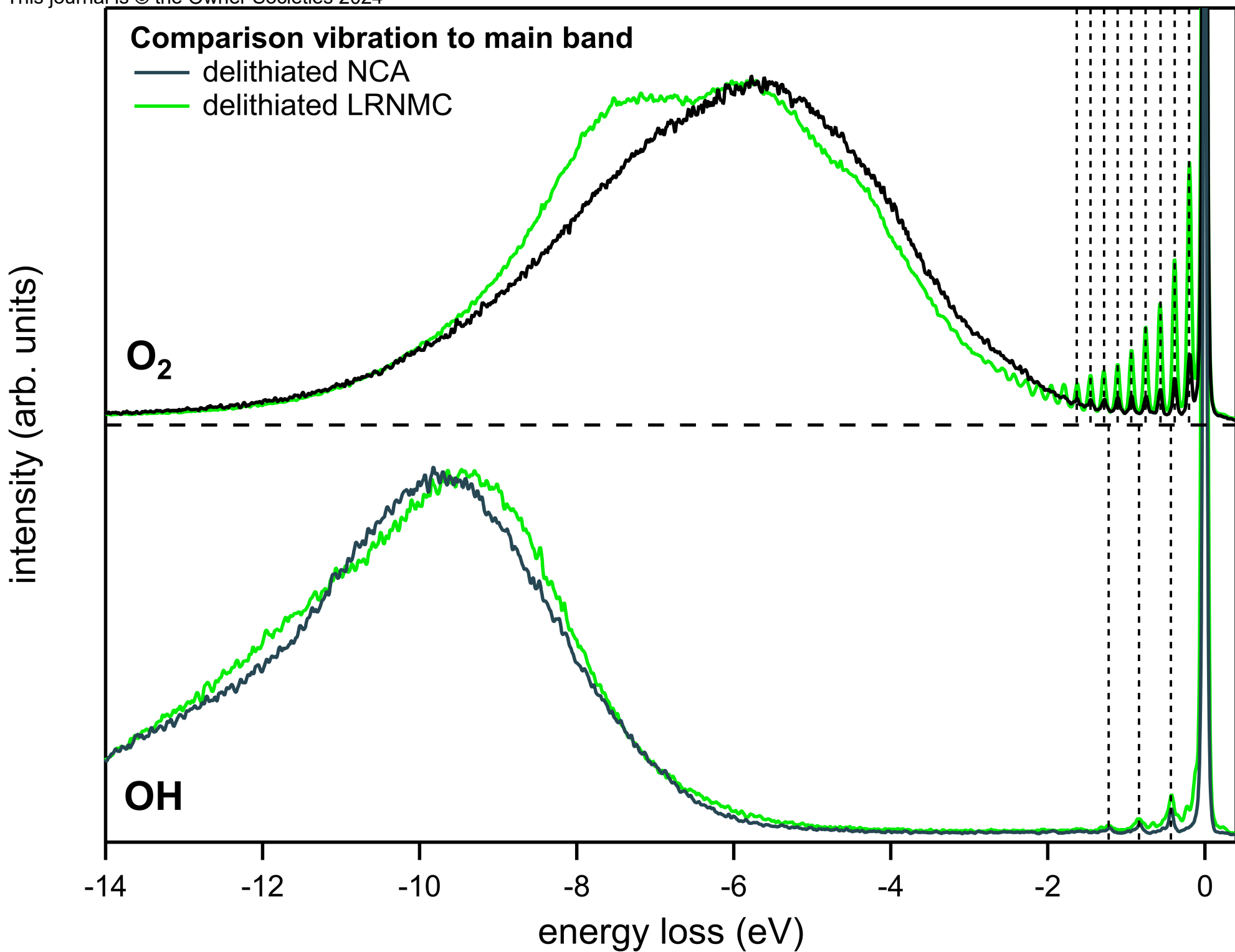

Supplement: CP-026-D4CP01766H-s006 [file CP-026-D4CP01766H-s006.pdf]

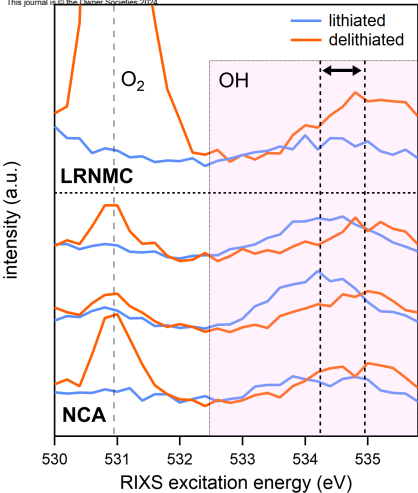

Supplement: CP-026-D4CP01766H-s007 [file CP-026-D4CP01766H-s007.pdf]
